# 

**Published:** 1827-10

**Authors:** 


### BIBLIOGRAPHICAL RECORD;

### OR,

*Works received for Review from the* 15*th of June to the* 15*th of September,* 1827.

1. A Dissertation on the Remote and Proximate Causes of Phthisis Pulmonalis; to which the Prize was adjudged for the year 1825, by the New York State Medical Society. By ANDREW HAMERSLEY, M.D. &c. Second Edition, revised and corrected, 12mo. pp. 99, New York, 1827.

☞ *We noticed the first Edition of this little Work with commendation, and the present edition is still more meritorious.*

---

2. Essay in Answer to the Questions proposed by the Government of the Duchy of Oldenburgh, respecting the Nature and Contagion of Yellow Fever. "In veritate spes mea est." Quarto pp. 32.

☞ *These are very able answers, and well deserved the prize which was offered, but we believe, never conferred.*

---

3. Introductory Lecture to the Course of Anatomy and Physiology, in Rutyer's Medical College, New York, delivered, November 11, 1826. By JOHN D. GODMAN, M.D. Professor of Anatomy and Physiology. 2nd Edition. 8vo. stitched, pp. 39, New York, 1827.

☞ *This is a very eloquent introductory lecture, from which we have inserted an extract in our Periscope, so applicable to the difficulties of studying anatomy in our own country.*

---

4. Observations on the Medical Character, Addressed to the Graduates of the College of Physicians and Surgeons of New York, at the Commencement, held on the 4th of April, 1826. By DAVID HOSACK, M. D. Vice President of the College, and Professor of the Theory and Practice of Physic and of Clinical Medicine. Published at the request of the Graduates. 8vo. stitched, pp. 38, New York, 1826.

☞ *An eloquent and excellent address.*

---

5. An Inaugural Discourse, delivered at the opening of Rutyer's Medical College, in the City of New York, on Monday, the 6th day of November, 1826. By DAVID HOSACK, M.D. F. R. S. &c. &c. &c. 8vo. pp. 176, New York, 1826.

---

6. Oratio Harveiana Prima in Novis Ædibus Collegii habita sext. Kalend. Jul. An. MDCCCXXVII. A. PELHAM WARREN, M.D. Col. Reg. Med. Lond. Necnon. Reg. Soc. Socio. Londini: Prostat. apud R. H. Evans, 93, Pall-Mall, MDCCCXXVII.

7. Lectures on the Operative Surgery of the Eye; or an Historical and Critical Inquiry into the Methods recommended for the Cure of Cataract, for the Formation of an Artificial Pupil, &c. &c. &c. containing a New Method of Operating for Cataract by Extraction, which obviates the Difficulties and Dangers hitherto attendant on that Operation; being the substance of that part of the Author's Course of Lectures on the Principles and Practice of Surgery, which relates to the Operations on the Eye and its Appendages. By G. J. GUTHRIE, F. R. S. Surgeon to the Westminster Hospital, &c. &c. &c. Second Edition. With seven Explanatory Plates. 8vo. pp. 555, London, 1827.

☞ *This Edition contains an immense mass of practical information, original and collected from various sources.*

---

8. Clinical Reports of the Medical Cases in the Meath Hospital and County of Dublin Infirmary, during the Session 1826, 1827. Part I. By R. I. GRAVES, M. D. and WILLIAM STOKES, M. D. Physicians to the Hospital. 8vo. stitched, pp. 83, two coloured lithographic plates. Dublin, 1827.

☞ *See Periscope of Hospital Reports in this Number.*

---

9. Annual Report on Diseases of the Eye, treated by GEORGE RICHMOND, Esq. Assist. Surgeon 4th Dragoons, and laid before the Bombay Medical Board.

☞ *We have transmitted this communication to one of our Cotemporaries, as we do not publish original papers. Mr. Richmond's zealous labours in the cause of humanity we noticed in a former Number of this Journal. The present report does great honour to the goodness of his heart as well as the dexterity of his hand.*

---

10. Die Behandlung der Lustseuche ohne Quecksilber oder die nicht merkuriellen Mittel und Methoden zur Heilung der Lustseuche. Nebsteinem kurzen Bericht uber die Anwendung der Antiphlogistiches Methode gegeu die se Kraukheit, im allegmeinen Kraukerharse zu Hamburg, von Dr. FRIEDRICH WILHELM OPPENHEIM, praktischem Arzte und Wundarzte in Hamburg. Hamburg, 1827.

11. Transactions of the Medical and Physical Society of Calcutta. Volume the Second. 8vo. pp. 430, Calcutta, 1826.

---

12. Medical Ethics; or, a Code of Institutes and Precepts, adapted to the Professional Conduct of Physicians and Surgeons. By the late THOMAS PERCIVAL, M.D. F. R. S. &c. &c. With additions illustrative of the past and present state of the Profession and its Collegiate Institutions, in Great Britain. 12mo. pp. 360, Jackson, London, 1827.

☞ *Mr. Jackson deserves thanks for republishing this excellent Work, at a time when Medical Ethics are much wanted, though little regarded. We shall dedicate an article to the Work shortly.*

---

13. Popular Lectures on the Study of Natural History and the Sciences, Vegetable Physiology, Zoology, the Animal and Vegetable Poisons, and on the Human Faculties, Mental and Corporeal, as delivered before the Isle of Wight Philosophical Society. By WILLIAM LEMPRIERE, M. D. Author of a Tour to Morocco; Observations on the Diseases of Jamaica, &c. &c. 8vo. pp. 304, London, 1827.

☞ *These Lectures not being addressed to the profession, we shall be unable to notice them farther than to say, that they are creditable to the author, and must prove very useful to all those who are about to dedicate a portion of their time to the useful purpose of diffusing scientific knowledge among the public at large.*

---

14. Introduction to the Science of the Pulse, as applied to the Practice of Medicine. By JULIUS RUCCO, M.D. Batchelor of Medicine in the Royal University of Naples; &c. &c. &c. Vols. II. royal 8vo. pp. 353-452. Burgess and Hill, London, 1827. Price 1*l*. 8*s*.

---

15. Morborum Definitiones causæque Continentes, &c. &c. Quibus accedit Toxicologia. Auctore RICARDO MADDOCK HAWLEY, M.D. Collegii Regii Medicorum Edinensis Socio, necnon Societatis Regiæ Medicæ Edinensis Socio Extraordinario. Edinburgi: apud Guthrie and Tait; necnon Londini. 8vo. pp. 364, 1827.

☞ *This work will prove highly useful to all those who have to undergo an examination in the Latin language, before any of the great medical tribunals of this country.*

---

16. A Synoptical Chart of the various Dislocations to which the Human Frame is subjected, comprising their Diagnostic Symptoms and Modes of Reduction; arranged by J. M. Cunningham, M. D. Second Edition. Price 3*s*.

☞ *This is no bad coup d' œil of the various accidents to which our joints are liable. We never saw the First Edition. The undertaking is ingenious ; and considering that the Chart occupies only* 18 *inches by* 14, *it contains a prodigious quantity of useful information respecting dislocations, which comes to the eye at a single glance. It is a useful thing in the surgery.*

---

17. No. VIII. of Medical Botany; or Illustrations and Descriptions of the Medicinal Plants of the London, Edinburgh, and Dublin Pharmacopœias, with those lately introduced into Medical Practice ; comprising their generic and specific Characters ; English, Provincial, and Foreign Appellations ; a Copious List of Synonymes ; Botanical Descriptions ; Natural History; Physical, Chemical, and Medical Properties and Uses : including also a Popular and Scientific Description of Poisonous Plants, particularly those that are indigenous to Great Britain and Ireland ; with Figures coloured from Nature ; the whole forming a complete System of Vegetable Toxicology and Materia Medica. By John Stephenson, M.D. of the University of Edinburgh, and James Morss Churchill, F. L. S. M. R. C. S. &c. &c. London, John Churchill. Price 3*s*. 6*d*. August 1, 1827.

---

18. Observations on Lithotomy, and on the Formation of Urinary Calculi. By John Charles Litchfield, Surgeon, Lecturer on Anatomy, &c. &c. &c. 8vo. pp. 48, London, 1826.

☞ *The object of the Author, and it is a very laudable one, is to draw the attention of surgeons more to the constitutional condition and treatment of their patients, than is the practice at present. His observations appear to be judicious, and we have reason to believe Mr. Litchfield a zealous and enterprising young man.*

---

19. The Necessity of Disinterment, under existing Circumstances, in a Letter to the Mayor of Exeter. By William Cooke, Surgeon. 8vo. stitched, pp. 31. 1827. Price 1s.

☞ *We have reverted to this little Essay in another place.*

---

20. Mems. Maxims, and Memoirs. By William Wadd, Esq. F.L.S. Surgeon Extraordinary to the King, &c. &c. &c. Quicquid agunt *Medici*, nostri est farrago libelli. 8vo. pp. 303, London, 1827.

---

21. The Medical and Surgical Student's Synopsis and Guide: comprising a detail of the proper Course of Study, and Works to be consulted, &c. &c. By G. T. Hayden, Licentiate of the Royal College of Surgeons in Ireland. 8vo. pp. 117. 1827.

☞ *This is a very useful Vade-Mecum for the Student—especially for him who studies in the Dublin school.*

---

22. Review of some Surgical Cases which have lately occurred in the Royal Infirmary of Edinburgh—a Clinical Lecture delivered to the Students of Surgery in that Institution on Thursday, 26th July, 1827. By George Ballingall, M.D. &c. &c. one of the Surgeons to the Royal Infirmary. 4to. pp. 24, with a Plate. Edinburgh, 1827.

☞ *These retrospective views will be useful, as the author observes, to those who have witnessed the different Cases. The sketches are too evanescent for others.*

---

23. Practical Observations on the Management and Diseases of Children. By the late Charles Thomas Haden, Esq. With Additional Observations, and a Biographical Notice of the Author, by Thomas Alcock, Surgeon. 8vo. pp. 192. London, September, 1827.

☞ *We hope to be able to give some account of this work in our next.*

---

24. The Philadelphia Monthly Journal of Medicine and Surgery. Edited by N. R. Smith, M.D. 8vo. pp. 56. No. 1, Vol. 1.

☞ *This new cotemporary has our best wishes for its success.*

---

25. A Treatise on Gun-shot Wounds, on Inflammation, Erysipelas, and Mortification, on Injuries of Nerves, and on the Wounds of the Extremities requiring the different Operations of Amputation; in which the various Methods of performing these Operations are shown, together with their After-treatment; and containing an Account of the Author's successful Case of Amputation at the Hip-joint, &c. &c. With five Explanatory Plates, &c.: being a Record of the Opinions and Practice of the Surgical Department of the British Army, at the termination of the Wars in Spain, Portugal, France, and the Netherlands, in 1814 and 1815. By G. J. Guthrie, F.R.S. Surgeon to the Westminster Hospital, &c. &c. Third Edition. September, 1827.

☞ *This highly interesting and greatly improved edition, we shall give account of in our next Number.*

---

26. Journal de Progrès des Sciences et Institutions Médicales en Europe, en Amerique, &c.: contenant, 1mo. Une Revue Médicale Etrangere; 2ndo. Une Repertoire Generale des Faits, Experiences, et Observations; 3tio. Une Serie de Monographies Originales sur les Diverses Parties de la Medicine, &c. Par une Association de Medecins. IVme. volume. 1827. 8vo. 288, royal octavo.

☞ *We have much pleasure in recommending this spirited and highly-talented Journal, which is already receiving great encouragement in America, as well as in Europe. The plan is nearly the same as that of our own Journal, and this interesting foreigner is well calculated to convey a good idea of the actual state of medical science in the different countries of Europe.*

N. B. We beg to inform the Editors that we have forwarded with this Number of our Journal, the Number for January 1827, according to their desire.

---

27. A Practical Treatise on the Diseases of the Skin, arranged with a view to their Constitutional Causes and Local Characters; and including the substance of the Essay on these Subjects, to which the Royal College of Surgeons awarded the Jacksonian Prize. 2nd Edit. corrected and enlarged. By Samuel Plumbe, M.R.C.S. 8vo. bds. pp. 470, with plates, Underwoods, September, 1827.

☞ *This Record closed on the 15th September.*

### LIST

OF

### SUCCESSFUL CANDIDATES

FOR

### THE HOSPITAL REPORT PRIZE.

Palmam qui meruit ferat.

| NO. | NAME. | HOSPITAL. | DATE. |
|---|---|---|---|
| 1 | Mr. Thos. H. Smith, (Pupil) | St. Thomas's. | April, 1827. |
| 2 | Mr. George Bury, (Pupil.) | Winchester. | July, 1827. |
| 3 | Mr. T. Fereday, M.R.C.S. | St. Bartholomew's | Octr. 1827. |
| 4 | Mr. C. W. Turner, (Pupil.) | Cheltenham. | Octr. 1827. |

Prize—*a complete Set of the* Medico-Chirurgical Review—*perpetual Registry of the successful Candidate's Name in the Journal.*

☞ The Hospital Reports are not confined to any particular period of time in the hospitals from which they are made. Facts are independent of dates. Candidates for the next Number are requested to forward their Reports by the 1st of December. Two prizes will be awarded next quarter—one metropolitan, one provincial.

*N. B.* The Editor having been from home when this Number of the Journal was closed, several Correspondents cannot be answered till next quarter. Dr. Brown's paper could not be found, and it is feared that it is lost. If he will forward a copy by the 1st of December, it will appear in the Extra-Limites, according to his desire. The List of Additional Subscribers is deferred till next number, when it will appear.

### INDEX.

A.

Abdominal tumours, some cases of 169
Abdominal inflammation, insidious case of ........ 489
Abscesses in the liver, unsuspected cases of ........ 278
Abscess of the liver, Dr. Graves's case of ........ 112
Abscess of the liver; a misnomer 522
Acupuncture........ 279
Acute tetanus, a fatal case of.... 232
Agenesis cerebral, cases of .... 420
Ague, Dr. Mackintosh on ...... 184
Alibert, M. on papular and crustaceous psorises ........ 189
Alum in diphtheritis, M. Bretonneau on........ 142
Amaurosis, case of ........ 279
Ammeline, Dr. his artificial preparations ........ 582
Amputation, during the progress of mortification ........ 219
Amputation for gangrene ...... 238
Amputation of a finger, followed by death ........ 264
Amputation of part of the lower jaw........ 289
Anchylosis, cured by a novel operation ........ 272
Andral, M. his clinique medicale 73
Andrews, Mr. on lunar caustic in stricture of the urethra....... 69
Aneurism of the innominata and subclavian ........ 533
Aneurism of the innominata, Mr. Wardrop's case........ 534
Aneurism of the abdominal aorta 233
Aneurism of the carotid, supposed case of ........ 271
Aneurism at the bend of the arm, Mr. White's case........ 277
Aneurismal tumour, rather anomalous case of........ 156
Aneurismal pouch in the heart, M. Cruveilheir's case........ 401
Aorta, obliteration of the ...... 426
Apyrectic diseases, Prof. Fulci on 225
Arnott, Dr. his elements of physics 35
Arnott, Dr., reply of Dr. Barry to 436
Arnott, Mr. on the contagious character of erysipelas ...... 148
Articulating cartilages, M. Cruveilheir on ........ 165
Articular rheumatism ........ 197
Artificial anus, curious case of .. 558
Arsenic and laudanum, poisoning by ........ 170
Arsenic in chorea, very large doses of........ .. 164
Arsenic, Dr. Christison on...... 379
Ascites with pregnancy, case of.. 409
Ascites, periodical, a case of .... 227
Asphyxia, M. Leroy on ........ 234
Asthma, M. Broussais on ...... 167
Astragalus, case of extraction of the........ 457
Atropa belladonna, its properties, &c........ 98
Aura electrica, remarkable case of ........ 459
Auscultation, mediate, Dr. Stack's inquiry into ........ 297
Axillary artery torn, a case of .. 452
Axillary nerves torn during the reduction of a luxation ...... 454

B.

Bally, M. his cases of obliterated arteries ........ 218
Baron, Dr. his "Life of Edward Jenner, M. D." ........ 102
Barton, Dr. his case of anchylosis 272
Bell, Mr., on strictures of the rectum ........ 427
Belladonna, case of poisoning by 233
Bile, effused into the substance of the liver........ 274
Bladder, curious case of perforation of the........ 438
Bladder, puncture of, Mr. Fereday........ 561
Bleeding in the cold stage of ague, Dr. Mackintosh on ........ 184
Brain, metastasis of rheumatism to 351
Brain and spinal marrow, diseases of the ........ 438

Breast, case of encysted tumour of the .................... 261
Breschet, M. on equivocal sanguineous tumours .......... 159
Bouillaud, Dr., on pulmonary apoplexy .................. 466
Bouillaud, Dr. his case of hypertrophy of the stomach........ 221
Bouillaud and Cruveilheir on the anterior lobes of the brain.... 129
Brodie, Mr. on poisonous matter in offal .................. 166
Brodie, Mr. his case of ununited fracture.................. 478
Bronchocele, successfully treated by seton .................. 263
Bronchotomy, for cynanche laryngea .................... 417
Broussais, his propositions of medicine .................... 46
Bursa of the knee-joint, affections of the ................ 500
Bury, Mr., his prize hospital report ...................... 249

C.

Cæcum, ruptured, Mr. Speer's case of .................... 121
Cancer of the penis............ 243
Carbonate of iron in neuralgia.. 403
Cardia, interesting case of cancer of ........................ 439
Cardiac veins, great dilatation of the.......................... 279
Carious joints, Dr. Crampton's cases of excision of.......... 301
Carotid aneurism, Mr. Lambert's case of .................... 214
Carotid aneurism, ligature beyond the tumour............ 209
Catarrho-rheumatic ophthalmia, Mr. Mackenzie on .......... 136
Cephalo-spinal fluid .......... 228
Cerebral agenesis, memoir on .. 420
Cervical vertebra fractured .... 240
Cervical vertebra dislocated, without fracture .................. 243
Cervix scapulæ, case of fracture of the........................ 267
Charter of the College ........ 509
Charles, King, his letter to the College of Physicians ........ 515
Cheyne, Dr. on a fatal erethism of the stomach.............. 124
Chorea, Mr. Turner on ........ 569
Chorea, Dr. Hawkins' fatal case of .......................... 146
Chorea, large doses of arsenic in 164
Christison, Dr., on arsenic...... 379
Chronic rheumatism, treatment of .......................... 352
Chronic enlargement of the testicle ........................ 529
Chronic diarrhœa, Dr. Elliotson on .......................... 155
Cicatrix, excision of, in hydrophobia........................ 236
Circular swing in insanity, its powers ...................... 29
Cleft palate, operation unsuccessful for .................... 522
Clinical assistants ............ 284
Cooke, Mr., *persecution* of ...... 286
Cooper, Sir A., on disease of the nails ........................ 181
College of Physicians .......... 505
Colles, Dr., on dissection wounds 306
Colica pictonum, Dr. Ranque on 474
Colica pictonum, M. Andral on.. 86
Colica pictonum, supposed case of, in the Royal Infirmary .... 92
Colica pictonum, cured by tobacco fomentations and croton oil .......................... 114
Commentaries on female diseases 316
Compound fracture of the cranium, two cases of .......... 195
Compound fracture of the skull, without depression .......... 525
Contagious psoriasis, supposed instances of ................ 116
Constipation, Dr. Crampton's case of .................... 118
Cranium and spine, fracture of the 485
Cruveilheir, M. aneurismal pouch in the heart................ 401
Cuming, Dr. on an affection of the mouth.................. 120
Cusack, Mr. on amputation of the jaw........................ 289
Cutaneous diseases, Drs. Graves and Stokes on .............. 479
Cynanche laryngea; tracheotomy, successful.................. 122

D.

Diabetes mellitus, M. Luroth's case .......................... 496
Dilatation of the urethra, M. Dupuytren on ................ 449
Diseased and wounded arteries.. 461
Diseases of the hip-joint, cases of 254
Disorder of the general health in females, chronic form........ 324
Dissection wound, fatal case of.. 306
Distention of the duodenum .... 366

Dropsy, various kinds and treatment of.................... 62
Dupuytren, M. his case of ligature on the external iliac .... 177
Dyspepsia, curious case of...... 412

E.

Earle, Mr., his cases of strangulated hernia ................ 472
Emphysema of the lung, case of 312
Emphysema, Mr. Fereday on.... 545
Empyema, surprising case of.... 491
Encephaloid disease of the liver 487
Epileptic lunatics, treatment of 30
Erethism of the stomach ...... 124
Ergot of rye, employment of.... 278
Erysipelas, contagious ........ 148
Erysipelas phlegmonodes, incisions in ...................... 280
Erysipelas after amputation .... 554
Erysipelas, death from incisions in 536
Erysipelas œdematodes, Mr. Fereday..................... 556
Erysipelas, treated by incisions.. 244
External iliac, ligature of ...... 177
Eye, case of deep-seated inflammation in the .............. 265

F.

Female youth, diseases of ...... 317
Fereday, Mr. prize hospital report 537
Fevers, local bleeding in......... 49
Fever, Dr. Bally on the seat of.. 498
Flaubert, M. on the reduction of dislocations ............... 452
Follicular ulceration, case of.... 490
Fracture, ununited, of the tibia 315

G.

Gall-bladder, case of inflammation of ..................... 187
Gastro-enterite, fatal case of.... 481
Gastro-enteritis, treatment of .. 57
Gastro-enteritis, without pain .. 458
Gastrotomy for strangulated intestine .. .................. 223
Gastrodynia, M. Barrut on .... 408
Gastric *excitation*.............. 80
Gold-headed cane, memoirs of .. 38
Glossitis cured by incisions .... 411
Green tea, Mr. Newnham on.... 143
Gregory, Dr., on vaccination .. 459
Groin, scirrhous testicle in the.. 239
Guthrie, Mr. on gun-shot wounds 398

H.

Hall, Dr. Marshall, on the diseases of females ............ 316
Hæmaturia, cured by the sound 145
Harrison, Dr., his circular on the College .................. 268
Heart, metastasis of rheumatism to the..................... 348
Helot, the contented .......... 583
Hepatic abscess, curious case of 135
Hepatic tumour, M. Luroth's case 186
Hernia, cases of ............... 521
Hernia, strangulated, cases of, with remarks .............. 523
Hernia, with retention of urine 275
Hiccup lasting 12 days, case of.. 196
Hip-joint, disease of the........ 254
Hip-joint, successful amputation of the..................... 418
Homœopathy, wonders of ...... 432
Hopkins, Dr. his obstetric apparatus ....................... 582
Hospital reports, observations on 584
Hunger, a cause of gastritis .... 52
Hydrophobia, some cases of .... 236
Hydrophobia, amputation of the limb in .................... 237
Hypertrophy of the heart ...... 199
Hysteria, various forms of...... 329

I.

Incisions in erysipelas ...... .. 244
Incontinence of urine, cured by dry cupping ................ 278
Indigestion, Dr. Philip on...... 362
Inflammation of the prepuce, with phymosis ................. 526
Inflammation of the spinal marrow ...................... 464
Insanity, pathology of.......... 17
Insipidity of the bile, remarks on 367
Intermittent inflammation....... 60
Intermittent rheumatic fever .. 171
Intermittent nasal hæmorrhage 196

J.

Jacob, Dr. on a peculiar ulcer of the eye-lids ............... 123
Jacobson, Dr. his anastomosis between the fifth and sympathetic 172
Jenner, Dr., his life, by Dr. Baron 102
Joints, carious, cases of excision of ........................ 301

K.

Keratonyxis for cataract ....... 208
Kino, effectual in a case of dropsy ....................... 134
Knee-joint, case of excision of the 303
Knight, Dr. on mental derangement...................... 1

L.

Labia pudendi, infiltration of .. 431
Lactuca virosa, its effect on the pulse ........................ 102
Lancet, review of hospital reports in ........................ 241
Larynx, ulceration of, Mr. Fereday ........................ 554
Lee, Dr. on the nutrition of the fœtus ........................ 504
Ligature of the pneumo-gastric nerves ........................ 410
Ligature of the common iliac artery ........................ 416
Ligature beyond the aneurismal tumour ........................ 209
Lithotomy, successful ........ 267
Lithotomy fatal, case of ....... 555
Lithotomy in an old person, fatal case ........................ 526
Lithotomy, fatal, some cases of, with remarks ............... 532
Lower jaw, amputation ........ 289
Lower jaw, compound fracture of 529
Lunar caustic in urethral stricture 69
Lunar caustic, in wounds, bruises, &c........................ 153

M.

Macilwain, Mr. on strictures.... 69
Mackenzie, Mr. on catarrho-rheumatic ophthalmia ...... 136
Madeira, its climate for phthisis 173
Mammary diseases, classification of ........................ 162
Medical botany ............... 95
Medical science, Dr. Shute's principles of................ 358
Mental diseases, Dr. Voison on the causes of .............. 1
Mercury, disease of the bones after ........................ 260
Mercurial frictions in puerperal peritonitis.................. 201
Midwifery, Sir A. Carlisle ...... 587
Mortification, amputation during its progress ............... 219
Mouth, a peculiar affection of .. 120
Mucous membrane of the stomach 55
Mucous membrane of the stomach, softening of the ...... 82
Musgrave, Dr, on the effects of mercury ........................ 423

N.

Nails, Sir A. Cooper on disease of the........................ 181
Nævi materni, *vaccination* in .. 280
Nævus maternus, treated by ligature ........................ 243
Nerves of sense and motion .... 200
Newnham, Mr. on green tea.... 143
Neuralgia spasmodica, Dr. Scudamore on ............... 355
Nitrate of potass in indigestion 376
Noxious exhalations, influence of 280
Nugæ Canoræ ................ 38
Nymphomania and satyriasis .. 16

O.

Obstruction of arteries, M. Bally on ........................ 218
Osteo-sarcoma of the jaw, Mr. Fereday........................ 565

P.

Paracentesis abdominis, during pregnancy ................ 409
Paracentesis thoracis, successful case of ................ 273
Pathology of fever, Dr. Hewett on the ................ 494
Pelvis, fracture of ............ 276
Penis, cancer of ............ 243
Phagedæna gangrenosa ........ 529
Phagedæna, Mr. Fereday on.... 551
Phagedænic ulceration ........ 245
Philip, Dr. on protracted indigestion ........................ 362
Philip, Dr. on indigestion ...... 362
Phlebitis, Mr. Fereday on ...... 537
Phlebitis, fatal cases of ........ 241
Phlebitis, fatal case of ........ 245
Physics, Dr. Arnott's elements of 35
Physicians, charter of the college of ........................ 505
Pneumonia without pain ...... 458
Pneumonia mistaken for hydrothorax ........................ 482
Poisoning by belladonna ...... 233
Poisoning by arsenic, Dr. Christison ........................ 379
Poisonous matter in offal ...... 166
Polypi, nasal, case of........... 265
Prize hospital reports, Mr. Fereday........................ 537
Prize hospital report, Mr. Turner 569
Prize hospital report, Mr. Bury.. 249
Proteian malady ............... 412
Proteian malady, M. Sarrut's own case ........................ 149
Protracted lactation, Dr. Morton on ........................ 488
Prurigo, cured by colchicum.... 529
Psoriasis, successful treatment of 479
Psoris papulosa, M. Alibert on .. 189

Psoris crustacea, M. Alibert on.. 191
Puerperal peritonitis .......... 201
Pulmonary apoplexy .......... 466
Pulsation in the veins, Dr. Davis's case of .................... 118
Purpura hæmorrhagica, cases of 468

R.

Radcliffe, Dr. memoir of ...... 39
Radial arteries, unequal pulse in 279
Reduction of dislocations, accidents following the ........ 452
Renton, Dr. on the dysentery of Madeira.................... 407
Rheumatism, Dr. Scudamore on 337
Rheumatism of the temporal muscles ..................... 113
Richard, Dr. case of hypertrophy of the heart ................ 465

S.

Salivation fatal, a case ........ 409
Sanguineous tumour, ligature of the artery for .............. 161
Sanguineous tumour, some anomalous cases of ............ 159
Section and ligature of the pneumogastric nerves............ 410
Scirrhous testicle in the groin .. 239
Scirrhous pancreas, M. King's case ...................... 493
Scirrhus of the stomach........ 73
Sciatica, Dr. Scudamore on .... 354
Scrotal hernia, fatal case of .... 246
Scudamore, Dr., on rheumatism 337
Seton in a case of bronchocele.. 263
Seton in cases of ruptured tendon 451
Shaw, Mr. obituary of ........ 581
Shute, Dr., principles of medical science .................... 358
Silk-worm gut ligature ........ 253
Sloughing chancre ............ 531
Spinal marrow, inflammation of the....................... 464
Spinal disease, fatal case of .... 247
Sponge-tent, as a dilator of the female urethra....... ..... 567
Steno-cardia, or angina pectoris 430
Stomach, effects of improper stimuli on .................... 50
Stomach affection, remarkable case of .................... 84
Stomach, hypertrophy of the.... 221
Strangulated intestine, gastrotomy for .................. 224
Strangulated hernia............ 472
Strangulated hernia, cases of.... 523
Strictures of the rectum, Mr. Bell on ....................... 427
Stricture of the urethra, Mr. Macilwain on....... ......... 69
Sulphate of copper in chronic diarrhœa ................ 156
Syphilis, communicated to a fœtus 244

T.

Taraxacum and stramonium, their properties.................. 100
Tartar emetic plaster in colica pictonum ................. 475
Tendo Achillis, rupture of the.. 451
Tetanus, acute, a fatal case of.. 232
Tetanus, M. Broussais on ...... 175
Thigh, elongation of ; a case.... 255
Thigh, shortening of ; cases of.. 257
Tibia, case of ununited fracture of 315
Tibia, aneurismal tumour in its interior ................... 156
Tic douloureux, severe case of .. 356
Tracheotomy, successfully performed ................... 122
Transfusion of blood, case of.... 415
Travers, Mr. on wounded arteries 188
Travers, Mr. on diseased arteries 461
Tubercular affections of the abdomen ..................... 334
Turner, Mr. prize hospital report 569

U.

Ulcer, peculiar, of the eye-lids.. 123
Ulcer of the tongue, curious case of ....................... 531
Urethra, M. Dupuytren on dilatation of .................. 449
Urine, passage of substances into 404
Uterus, inflammatory affections of ....................... 335
Ununited fractures, pressure in 478

V.

Voison, Dr. on the causes of mental diseases ............ 1

W.

Wadd, Mr. his mems. maxims, &c. ...................... 393
Wardrop, Mr., his case of carotid aneurism ................ 209
Whitish stools, or fluxus cœliacus 114
Winchester hospital report .... 249
Wounded arteries, cases of...... 188
Wound of the "brachial vein".. 527
Wound of the knee-joint ...... 528
Wound of the larynx and œsophagus ... ................ 531

Y.

Yellow fever, bleeding and abstinence in ................. 54

